# Identification of a Novel Intronic Mutation in *VMA21* Associated with a Classical Form of X-Linked Myopathy with Autophagy

**DOI:** 10.1055/s-0044-1786815

**Published:** 2024-05-10

**Authors:** Mainak Bardhan, Kiran Polavarapu, Dipti Baskar, Veeramani Preethish-Kumar, Seena Vengalil, Saraswati Nashi, Valakunja H. Ganaraja, Dinesh Sharma, Karthik Kulanthaivelu, B.N. Nandeesh, Atchayaram Nalini

**Affiliations:** 1 Department of Neurology, National Institute of Mental Health and Neuro Sciences (NIMHANS), Bengaluru, Karnataka, India; 2 Children's Hospital of Eastern Ontario Research Institute, Ottawa, K1H 5B2, Ontario, Canada; 3 Deaprtment of Neuroradiology, National Institute of Mental Health and Neuro Sciences (NIMHANS), Bengaluru, Karnataka, India; 4 Department of Neuropathology, National Institute of Mental Health and Neuro Sciences (NIMHANS), Bengaluru, Karnataka, India

**Keywords:** *VMA21* -related myopathy, autophagy, muscle MRI

## Abstract

**Introduction** *VMA21* -related myopathy is one of the rare forms of slowly progressive myopathy observed in males. Till now, there have been only a handful of reports, mainly from Europe and America, and two reports from India.

**Method** Here, we describe a case of genetically confirmed *VMA21* -associated myopathy with clinical, histopathological, and imaging features with a list of known VMA21 mutations.

**Results** A 29-year-old man had the onset of symptoms at 18 years of age with features of proximal lower limb weakness. Muscle magnetic resonance imaging showed the preferential involvement of vasti and adductor magnus. A biopsy of the left quadriceps femoris showed features of autophagic vacuolar myopathy with vacuoles containing granular eosinophilic materials. In targeted next-generation sequencing, hemizygous mutation in the 3′ splice site of intron 2 of the *VMA21* gene (c.164–7 T > A) was identified and confirmed the diagnosis of X-linked myopathy with excessive autophagy.

**Conclusion** This report expands the phenotypic and genotypic profile of *VMA21* -related myopathy, with a yet unreported mutation in India.

## Introduction

X-linked myopathy with excessive autophagy (XMEA) is one of the early-onset myopathies that predominantly affects males. 1 It results mainly from a genetic defect in *VMA21* leading to alteration in chaperone assembly of vacuolar ATPase and increases the lysosomal pH, 2 hence also called as *VMA21* -related myopathy. Histopathologically, it is characterized by autophagic vacuoles with sarcolemmal features. 3 The onset of symptoms is usually as early as in the second decade, with variable severity. 4 The features of *VMA21* -related myopathy closely resemble those with congenital autophagic vacuolar myopathy (CAVM); however, clinical presentation is largely confined to term neonates with respiratory failure requiring ventilatory support. 5 There are fewer than 50 cases reported worldwide. Here, we present the clinical, radiological, histopathological, and genetic features of a young man with XMEA from India.

## Case History

**Fig. 1:**
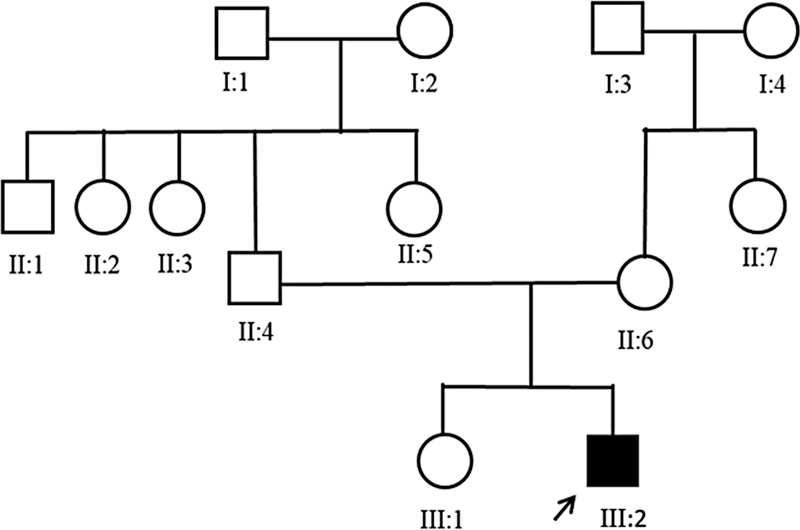
Pedigree chart of the patient.

A 29-year-old man born from nonconsanguineous parents was evaluated in 2019. He presented with gradually progressive weakness of lower limbs from 18 years of age. Initially, he noticed difficulty while cycling and running fast. Over the next year, he started experiencing buckling episodes and falls while walking. By 25 years of age, he developed difficulty rising from the floor and climbing stairs. There was no feature of distal limb weakness, myalgia, cramps, contractures, or cardiac symptoms. There was no significant positive family history ( Fig. 1 ).

On examination, he has mild calf hypertrophy. The muscle power, according to modified Medical Research Council grading, was as follows: infraspinatus (4), Iliopsoas (4), hip adductors (4 + ), quadriceps (4), and the rest of the power examination was normal. Tendon reflexes were normal except for hypoactive knee jerks. There was no scapular winging or muscle contractures. The creatine kinase level was 6630 IU/L (normal: 20–200 U/L). The electrocardiogram and two-dimensional echocardiography were normal. Nerve conduction study was normal.

**Fig. 2:**
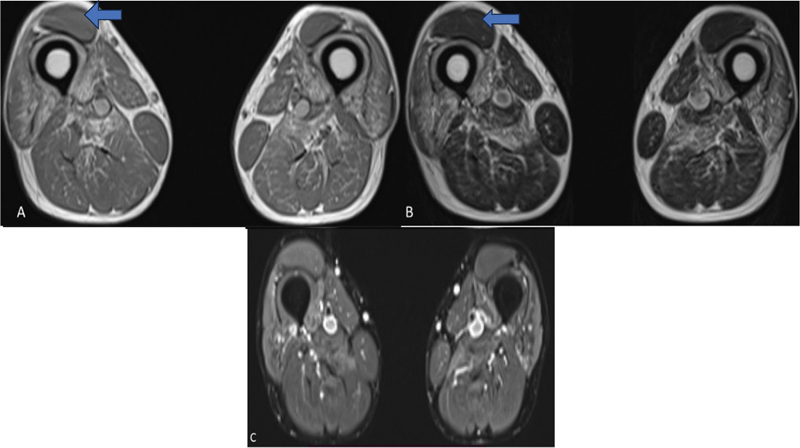
Muscle MRI of the patient. ( **A** ) T1W, ( **B** ) T2W, and ( **C** ) STIR sequences axial sections in the midthigh region demonstrate fatty atrophy and volume loss of bilateral vastus medialis, intermedius, and lateralis with involvement of adductor magnus. Rectus femoris is relatively spared ( *arrow* ). In the bilateral thigh muscles, there was no aberrant STIR signal intensity.

Muscle magnetic resonance imaging (MRI) showed bilateral symmetric volume loss with fatty replacement of the proximal thigh muscles, preferentially involving the vasti and adductor magnus with relative sparing of the rectus femoris. In the thigh muscles, there was no aberrant short tau inversion recovery signal intensity ( Fig. 2 ).

**Fig. 3:**
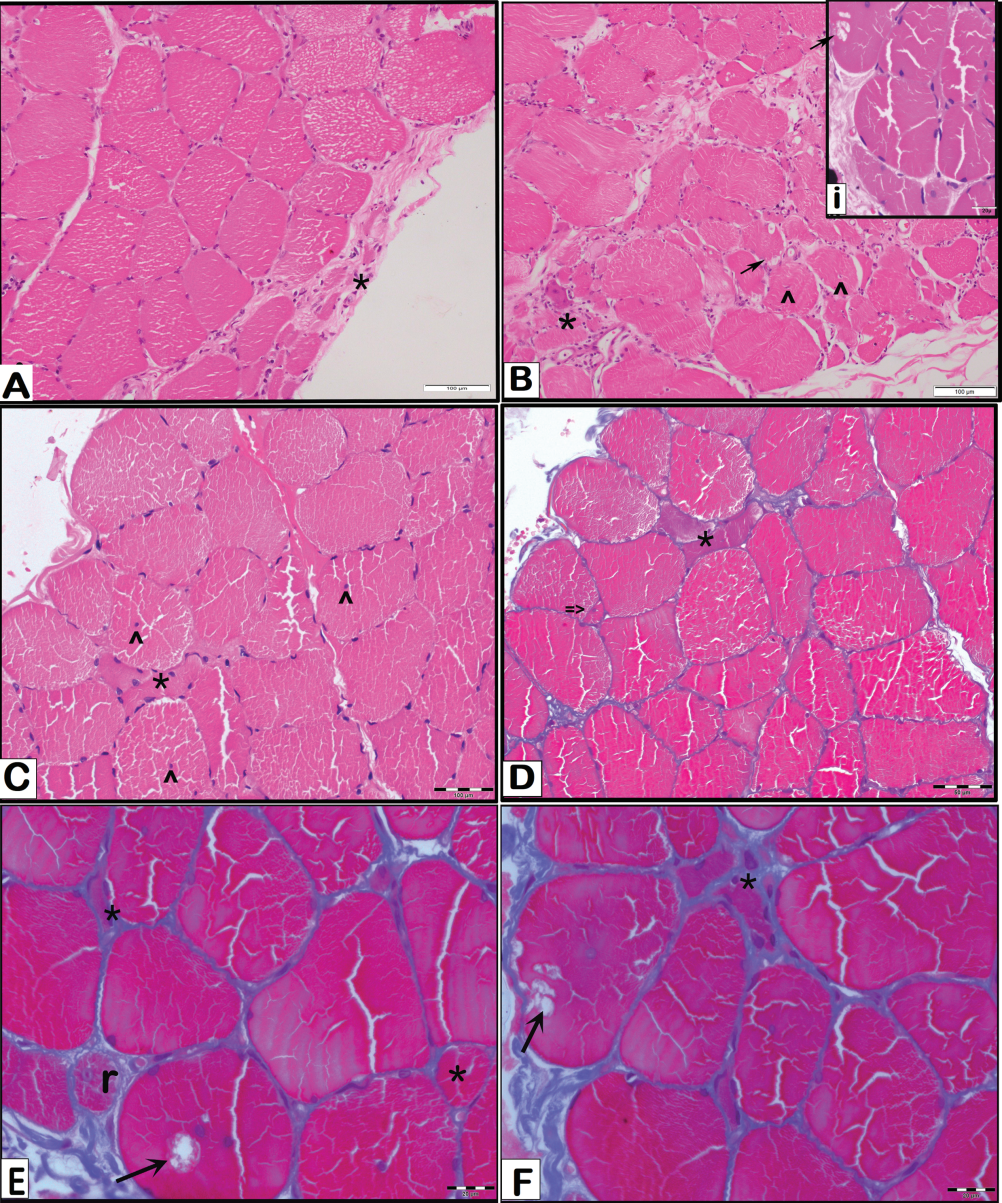
Histopathology of muscle biopsy. ( **A,B** ) Microphotograph showing a transverse section of skeletal muscle with variation in fiber size including atrophic angulated fibers (asterisk *), internalized nuclei (^) and a few hypertrophic fibers. (H&E stain; scale bar A, B—100 µm) Inset (i) shows a vacuole in one of the myofibers (H & E, scale bar—20 µm). ( **C–F** ) Microphotograph showing a transverse section of skeletal muscle with variation in fiber size including atrophic angulated fibers (asterisk, *) and a few hypertrophic fibers. A few of the fibers show vacuoles with granular eosinophilic material within ( *arrows* ). Internalized nuclei (^) and a regenerating fiber (r) are observed. Occasional fiber shows splitting (≥). Also note the interstitial/endomysial fibrosis as evident in E and F. (Fig C—H & E, scale bar 50 µm; D, E, F—Masson's trichrome stain, scale bar: D—50 µm; E, F—20 µm).

Open muscle biopsy was done from the left quadriceps femoris muscle and analyzed using hematoxylin and eosin and Masson's trichrome stains. Histopathology showed fiber size variation with scattered atrophic angulated fibers and a few hypertrophic fibers, along with internalized nuclei and splitting. A few of the fibers also showed vacuoles containing granular eosinophilic material within it ( Fig. 3 ).

**Fig. 4:**
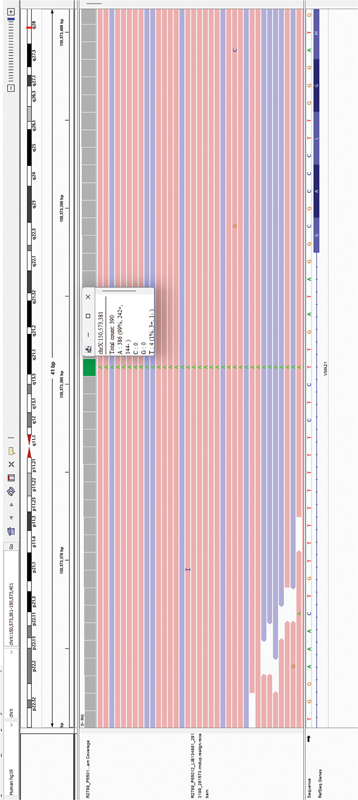
Integrative genome viewer screenshot of the c.164–7T > A variant.

Exome sequencing showed hemizygous mutation in the *VMA21* gene (chrX:g.150573381T > A, hg19) affecting the seventh nucleotide position upstream of exon 3 (c.164–7T > A; ENST00000330374.6) in the 3′ splice site of intron 2 ( Fig. 4 ). In-silico analysis using spliceAI ( https://spliceailookup.broadinstitute.org/ ) predicted loss of canonical acceptor site at intron2/exon3 junction (Δ score: 0.39; threshold ≥ 0.2). 6

## Discussion

Our patient had onset of symptoms in the second decade with features of indolent proximal lower limb involvement and imaging features suggesting preferential involvement of adductor component of thigh muscles and also classical histopathological findings with next-generation sequencing confirming the diagnosis. Though there are many case reports from Europe and America, there are only a few cases from Asia (Comparison with previous studies is given in Table 1 ). 1 3 4 5 7 8 9 10 11 12 13 14 15 16 17

**Table 1:** Comprehensive details of salient features of current patient and published studies

S.no	Authors/ year	Ethnicity/ total no. of patients included	Age at onset (years)	Age at presentation (years)	Clinical presentation	Disease course	Consanguinity/ family history	Muscle biopsy	Muscle MRI	Genotype features
1	Rajeshwari et al/ 2022 7	Indian/1	6	8	Proximal lower limb weakness	Slowly progressive	−/−	Fiber size variation with autophagic vacuoles and MAC deposits.	–	Single nucleotide substitution at the splice branch point of intron1 (X:150572076A> T; c. 54–27A> T)
2	Rao et al 2019 8	Indian/1	Early childhood	5	Delayed motor milestones with proximal lower limb and facial weakness	Slowly progressive	−/−	Myopathic changes with autophagic vacuoles	–	–
3	Crockett et al 2014 3	American/1	55	65	Proximal lower limb weakness	Slowly progressive	−/+ (elder brother with limb weakness since mid-40s and wheel chair bound by lates 50's.)	Numerous cytoplasmic vacuoles staining positively for extracellular matrix protein Perlecan but negative for collagen VI Large, debris-containing intracellular vacuolar inclusions that focally disrupted sarcomeric structure of the muscle fibers representing autophagic vacuoles.	–	Substitution of a pyrimidine into a purine, c.164–7T > G
4	Alon et al 2021 9	Jewish Israeli/1	20	25	Proximal lower limb weakness	Slowly progressive	+/Maternal grandfather (onset-20 y) and male cousin (10 y)	Autophagic vacuoles and MAC deposits.	Fatty replacement of the vastus lateralis, semimembranosus, and gluteus maximus muscles	hemizygous mutation c.272G *> C* on the 3rd exon
5	Ruggieri et al 2015 10	I: Italian, II: European-Australian/2	I: birth II: early childhood	I: 14 II: 21	I: Hypotonia with respiratory distress followed by reduced muscle bulk in upper limbs and thoracic muscles with febrile seizures. II: delayed milestones followed by proximal upper and lower limb weakness.	Slowly progressive	I: −/− II: −/maternal uncle (onset: childhood)	I & II: Markedly variable fiber size. Increase in endomysial and perimysial fat and fibrous connective tissue, Multiple basophilic vacuoles in numerous fibers.	−	I: 92 bp microdeletion, c.*13_*104del, in 3′UTR. II: 9 bp microdeletion, c.54–16_54–8del, upstream of exon 2
6	Cotta et al 2020 11	Brazilian/2	I: 4 II: 7	I: 7 II: 20	I: Proximal lower limb weakness II: Proximal lower limb weakness	Slowly progressive	I: −/four male cousins, maternal uncle, II: −/−	I & II: Intrasarcoplasmic autophagic vacuoles with sarcolemmal features autophagic vacuoles	I: Adductor magnus, preferential peripheral vastus lateralis involvement, partial Rectus femoris, and peripheral Tibialis anterior and soleus muscles involvement. II: Adductor magnus, superficial vastus lateralis, peripheral rectus femoris.	I: c.163 + 4A > G II: c.272G > C
7	Pegat et al 2022 12	French/1	11	16	Tiptoe walking followed by proximal lower limb weakness	Slowly progressive	−/−	Numerous cytoplasmic autophagic vacuoles strongly positive for PAS and phosphatidic acid. p62 and TDP 43) were also positive in cytoplasmic autophagic vacuoles.	Fatty infiltration of both anterior and posterior thigh.	Unreported, intronic, single-nucleotide substitution c.164–20T > A.
8	Blanco-Arias et al 2023 13	Spanish/4	At birth	One died during first hour of life, other three in second and third decades of life.	Severe muscle weakness with respiratory failure and death in early childhood	Rapidly progressive	−/all four patients from same family	Autophagic vacuoles and MAC deposits.	–	c.294C > T (Gly98 = )
9	Chabrol et al 2001 14	French/ 5 families – 8 patients	F-1: early childhood F-2: early childhood F-3: 12 y F-4: birth F-5: birth	F-1: 5 F-2: 5 F-3: 17 F-4: 3 F-5: 5	F-1: generalized fatigue. F-2: proximal lower limb weakness. F-3: proximal lower limb weakness. F-4: persistently elevated CK. F-5: infantile hypotonia followed by proximal lower limb weakness with a rigid spine.	F-1: nonprogressive. F-2: slowly progressive. F-3: slowly progressive. F-4: nonprogressive.	F-1: −/− F-2: −/− F-3: −/+ F-4: −/− F5: −/−	Increased variation in muscle fiber size, round atrophic fibers. Vacuoles were present.	–	–
10	Kalimo H et al 1988 1	Finnish/3	P-1: 5 P-2: 6 P-3: childhood	P-1: 13 P-2: 8 P-3: not examined clinically	All proximal lower limb weakness	P-1: slowly progressive. P-2: slowly progressive. P-3: NA	−/all three patients from the same family	–	–	–
11	Villanova M et al 1995 15	French/4	P-1: childhood P-2:18–19 P-3: 20 P-4: second decade	P-1: 25 P-2: 56 P-3: not examined P-4: 64	All proximal lower limb weakness	All slowly progressive.	−/ all four patients from same family	P-1: Increased variation in muscle fiber size, round atrophic fibers. Vacuoles were present. P-2: Increased variation in muscle fiber size, round atrophic fibers. Vacuoles were present. P-3: ND P-4: ND	–	–
12	Kurashige et al 2013 16	Japanese/3	F-1: 6 F-2: childhood F-3: childhood	F-1: 52 F-2:43 F-3:47	All proximal lower limb weakness	All slowly progressive.	F-1: −/two maternal uncles F-2 is maternal uncle of F-3	F-1: Muscle fibers with vacuoles containing delicate basophilic debris F-2: Muscle fibers with vacuoles containing delicate basophilic debris F-3: ND	F-1: Atrophy and fatty degeneration of femoral muscles, Rectus femoris spared F-2: ND F-3: ND	F-1: Hemizygous c.164–7T > G mutation F-2: ND F-3: ND
13	Mercier et al 2015 4	French/4	F-1: 12 F-2: 7 F-3: 18 F-4: third decade	F-1: 43 F-2: 14 F-3: 31 F-4: 33	F-1: Proximal lower limb weakness F-2: Toe walking F-3: Proximal lower limb weakness F-4: Proximal lower limb weakness	All slowly progressive.	F-1: −/− F-2: −/nephew F-3 and F4 are brothers.	F-1: Moderate myopathic pattern, with irregular fiber size, fiber splitting, and internal nuclei, Small vacuoles were observed. F-2: ND F-3: Moderate myopathic pattern, with irregular fiber size, fiber splitting, and internal nuclei, Small vacuoles were observed. F-4: Moderate myopathic pattern, with irregular fiber size, fiber splitting, and internal nuclei, Small vacuoles	F-1: Diffuse fatty degeneration in all muscles with more severe involvement in lower than in upper limbs and in proximal than in distal regions. F-2: Early fatty degeneration present in the pelvic girdle. F-3: Prominent involvement of the pelvic girdle and almost all thigh muscles. Shoulder girdle and proximal upper limbs are also affected. F-4: ND	F-1: intronic mutation (c.163 + 3A > G). F-2: intronic mutation (c.163 + 3A > G). F-3: intronic mutation (c.163 + 3A > G). F-4: ND
14	Munteanu et al 2016 5	Japanese/1	2 mo	–	Floppy infant	NA	−/−	Electron-dense-debris-filled vacuoles, deposition of complement membrane attack complex on vacuoles, and multiplication of the basal lamina	–	c.164–6T > G mutation
15	Fernandes et al 2020 17	Brazil/1	2	5	Distal lower limb weakness with electrocardiogram showed incomplete right bundle branch conduction	Slowly progressive.	−/Five males affected	presence of vacuoles in muscle fibers, as observed by the basophilic inclusions	–	c.54–30_54–27delinsT variant
16	Our study/ 2023	Indian/1	18	29	Proximal lower limb weakness	Slowly progressive.	−/−	Variation in fiber size including atrophic angulated fibers and a few hypertrophic fibers along with internalized nuclei. Few of the fibers also showed vacuoles with granular eosinophilic material within it.	Diffuse fatty atrophy and volume loss of bilateral anterior compartment of thighs with relative sparing of medial compartment muscles.	Hemizygous splice variation c.164–7T > A

XMEA affects males and has a slowly progressive proximal limb muscle weakness and normal cardiac function with onset ranging from childhood to adulthood. 10 VMA21 is an assembly chaperone for the principal mammalian proton pump required for lysosome acidification. Due to the genetic defect in *VMA21* , alteration in lysosomal acidification leads to a block in steps of macroautophagy and accumulation of autophagosomes. This results in vacuole formation filled with debris in the muscles. 2 These vacuolar changes can also be observed in Danon's disease; however, calcium deposition, basal lamina reduplication, and absence of cardiomyopathy suggest XMEA. 1 18

Most *VMA21* mutations have been reported to affect normal splicing, predominantly occurring in the introns 1 and 2. One missense, c.272G > C, and another synonymous mutation, c.294C > T, while present in exon 3 have also been shown to affect splicing ( 12 13 ). Mis-splicing mutations lead to the reduction of mRNA and, in turn, reduced expression of VMA21 protein. 2 Clinical phenotype severity has been described as directly related to the extent of mRNA expression reduction. 12 13 Intronic *VMA21* mutations affecting the polypyrimidine tract in intron 2 adjacent to the acceptor site (c.164–6T > G; c.164–7T > G; c.164–20T > A) have been reported in multiple patients. 3 5 16 The mutations -6T > G and -20T > A pyrimidine-to-purine substitutions have been associated with severe phenotypes of congenital onset CAVM and childhood onset with relatively severe progression, respectively. 5 12 In comparison, c.164–7T > G has been reported with milder XMEA phenotype with variable onset ranging from childhood to late adulthood (>50 years). 3 16 The pyrimidine-to-purine substitution at -7 position in intron 2 has been described to reduce the binding efficacy of the U2AF splice factor, causing abnormal splice recognition at the acceptor site. 2 Our patient also had a novel pyrimidine-to-purine change of T > A at the same -7 position reported in earlier XMEA patients. While we could not check the mRNA expression levels in our patient, based on in-silico predictions, we presume a similar impact as in previous patients with -7T > G substitution. This correlated with our patient's milder XMEA phenotype with second-decade onset.

The age of onset is quite variable, though typically described as childhood onset. There are various reports on adult-onset myopathy, as noted in our patient, 4 9 15 with the oldest being onset in the seventh decade, as reported by Crockett et al. 3 It typically affects males and spares carrier females. The phenotypic spectrum extends from floppy infant delayed milestones to proximal limb-girdle and respiratory muscle weakness. Usually, extra skeletal muscle involvement does not occur. However, Fernandes et al described a patient with childhood-onset distal lower limb weakness with incomplete right bundle branch block. 17

The earliest and most frequently affected muscles in XMEA are the pelvic girdle and proximal thigh muscles, with the anterior compartment more involved than the posterior compartment, mimicking limb girdle muscular dystrophy pattern. MRI pattern of muscle in XMEA typically shows early involvement of the vasti, adductor magnus, and soleus with relative sparing of the rectus femoris and tibialis anterior. 5 Although muscle imaging shows thigh involvement in almost all patients, few patients show preferential involvement of the adductor group. 1 16 However, selective involvement of peripheral vastus lateralis, partial rectus femoris, and peripheral tibialis anterior muscle has also recently been described. 11 Our patient also had typical findings as described in previous reports with fatty infiltration of the vasti and adductor magnus and sparing of rectus femoris, showing that imaging pattern could be a strong indicator toward XMEA diagnosis in clinically suspected patients.

Histopathological analysis of skeletal muscle may show muscle fibers with sarcoplasmic vacuoles containing degraded organelles, debris, and calcium crystals. These vacuoles can be seen within the muscle fibers' interior depths or superficially close to the sarcolemma. 18 Generally, inflammation, necrosis, or apoptosis are not observed. Instead, myofiber demise occurs through a novel form of autophagic cell death characterized by giant autophagic vacuoles, 2 to 10 µm in size, encircling sections of cytoplasm, including organelles. These vacuoles contain lysosomal hydrolases with incomplete contents of digestion. Definitive diagnosis includes clinical presentation and genetic mutation identification.

## Conclusion

Here, we report an Indian case of *VMA21* -related myopathy that adds to our knowledge of XMEA phenotype-genotype spectrum. The pathological features and clinical spectrum of XMEA are large and overlap with other disorders. It also highlights the preferential involvement of thigh muscles with distinctive sparing of rectus femoris, which may aid in the diagnosis of this rare myopathy.
